# What are we to think when results from mouse research contradict those from human experiments and clinical practice?

**DOI:** 10.1038/nutd.2016.31

**Published:** 2016-08-15

**Authors:** G Schofield, G Henderson, C Crofts, S Thornley

**Affiliations:** 1 Human Potential Centre, Auckland University of Technology, Auckland, New Zealand

Dear Sir,

The recent paper by Lamont *et al.*^1^ supplies evidence that does not support its conclusion that low carbohydrate high fat (LCHF) diets should be avoided in the treatment of diabetes or pre-diabetes. The diets in this experiment were poorly controlled for diet quality, the LCHF diet of highly refined ingredients such as cocoa butter, casein and sucrose being compared with a low fat chow diet supplying such recognizable foods as yeast, molasses, skim milk powder, wheat, fish meal and soybean paste. The animals used, New Zealand Obese (NZO) mice, have a phosphatidylcholine transfer protein (PCTP) defect never found in humans, resulting in increased hepatic fatty acid uptake and triglyceride accumulation.^2^ Only the male NZO mouse, as studied by Lamont *et al.*, is prone to diabetes, which is not the case in humans. Thus, the NZO mouse can supply information about the effects of obesity, but cannot answer the question of whether LCHF diets will cause diabetes in humans. It is notable that Lamont *et al.* did not discuss the special features of the NZO mouse model, nor the differences between the mouse diets and recommended human LCHF diets, before extrapolating the results of their experiment to humans.

The reference 26 cited by Lamont *et al.*, Kluth *et al.*'s^3^ NZO mouse study, is an important experiment, in which the diets were well-controlled for quality, with results which suggest a ‘two hit' cause of type-2 diabetes, whereby lipotoxicity from ectopic fat accumulation (‘first hit') sensitizes the pancreas to glucotoxicity from post-prandial hyperglycemia (‘second hit').

These high post-meal serum glucose levels are themselves, in humans, likely to be the result of insulin resistance due to the earlier accumulation of ectopic fat in the liver and pancreas.^4^ We suggest, therefore, that the optimum diet for treating diabetes would both minimize the risk of post-prandial hyperglycemia, and stimulate the mobilization of ectopic fat. Studies which indicate that LCHF diets are more effective at achieving these goals in humans than standard dietary approaches, and do not produce the deleterious effects seen in NZO mice, were cited in Lamont *et al.* In the current absence of magnetic resonance imagery evidence regarding the effect of LCHF diets on pancreatic fat in humans, other human studies, showing the beneficial effects of both hypocaloric^5, 6^ and *ad lib*^7, 8^ LCHF dietary approaches on nonalcoholic fatty liver disease, a disease of ectopic fat accumulation closely associated with type-2 diabetes, should also in our opinion have been included in the paper by Lamont *et al.* and informed its conclusions. Contrary to the gloomy predictions of Lamont *et al.*, a 12-month clinical study by Maekawa *et al.*^9^ shows that LCHF dietary advice, which is associated with good adherence, delays or reverses the progression of pre-diabetes to diabetes, and in many subjects also reverses impaired glucose. Subjects (*n*=36) in this study were given a reduced calorie target during a 7-day in-ward education period, but were instructed not to restrict calories or fat during the rest of the 12-month period.

In the pre-insulin era, Newburgh conducted a series of clinical investigations at the University of Michigan Medical School that confirmed the safety of the LCHF dietary approach to diabetes management. We feel that it is appropriate here to repeat Newburgh's 1929 censure of Elliott Joslin after Joslin had claimed, on insufficient evidence, that Newburgh's approach would prove harmful. The unfounded conclusions of Lamont *et al.*, and the widespread publicity given to their criticisms of LCHF diets, amount to ‘an unjustifiable interference with a method that is working well'.^10^
